# A review of the current state of research on artificial blue light safety as it applies to digital devices

**DOI:** 10.1016/j.heliyon.2022.e10282

**Published:** 2022-08-15

**Authors:** Nikita A. Wong, Hamed Bahmani

**Affiliations:** a Dopavision GmbH, Berlin, Germany; b Department of Physiology of Cognitive Processes, Max Planck Institute for Biological Cybernetics, Tuebingen, Germany

**Keywords:** Blue light, Safety, Eye, Retina, Light emitting diode, Circadian rhythm

## Abstract

Light is necessary for human health and well-being. As we spend more time indoors, we are being increasingly exposed to artificial light. The development of artificial lighting has allowed us to control the brightness, colour, and timing of our light exposure. Yet, the widespread use of artificial light has raised concerns about the impact of altering our light environment on our health. The widespread adoption of personal digital devices over the past decade has exposed us to yet another source of artificial light. We spend a significant amount of time using digital devices with light-emitting screens, including smartphones and tablets, at close range. The light emitted from these devices, while appearing white, has an emission spectrum with a peak in the blue range. Blue light is often characterised as hazardous as its photon energy is higher than that of other wavelengths of visible light. Under certain conditions, visible blue light can cause harm to the retina and other ocular structures. Blue light can also influence the circadian rhythm and processes mediated by melanopsin-expressing intrinsically photosensitive retinal ganglion cells. While the blue component of sunlight is necessary for various physiological processes, whether the low-illuminance artificial blue light emitted from digital devices presents a risk to our health remains an ongoing area of debate. As technological advancements continue, it is relevant to understand how new devices may influence our well-being. This review examines the existing research on artificial blue light safety and the eye, visual performance, and circadian functions.

## 1 Introduction

Artificial light is pervasive in modern society, illuminating the places where we spend a large part of our time. We spend around 90% of our day indoors, and much of our exposure to artificial light is from the lamps we use to light these spaces [1, 2]. Compared to natural outdoor light, artificial light is often much dimmer and has a different spectral distribution than sunlight. The illuminance of outdoor light can reach values of up to 130,000 lux depending on the location, weather, and elevation. Even on a cloudy day (e.g., 15,000 lux [3]) the illuminance of outdoor light is several orders of magnitude greater than that of indoor lighting, which is typically less than 1,000 lux [4, 5]. By its nature, artificial lighting also exposes us to light at times of day when we normally would not receive light from the sun [6]. Over the course of human evolution light has had an important influence on physiological processes, and light is necessary for our overall health and well-being [7]. The widespread adoption of electric lighting has significantly altered the brightness, spectral distribution, and timing of our light exposure relative to that of our pre-modern societies. This has had a measurable effect on our circadian system [8] and concerns about the effects of artificial light on human health are not new.

**Figure 1:**
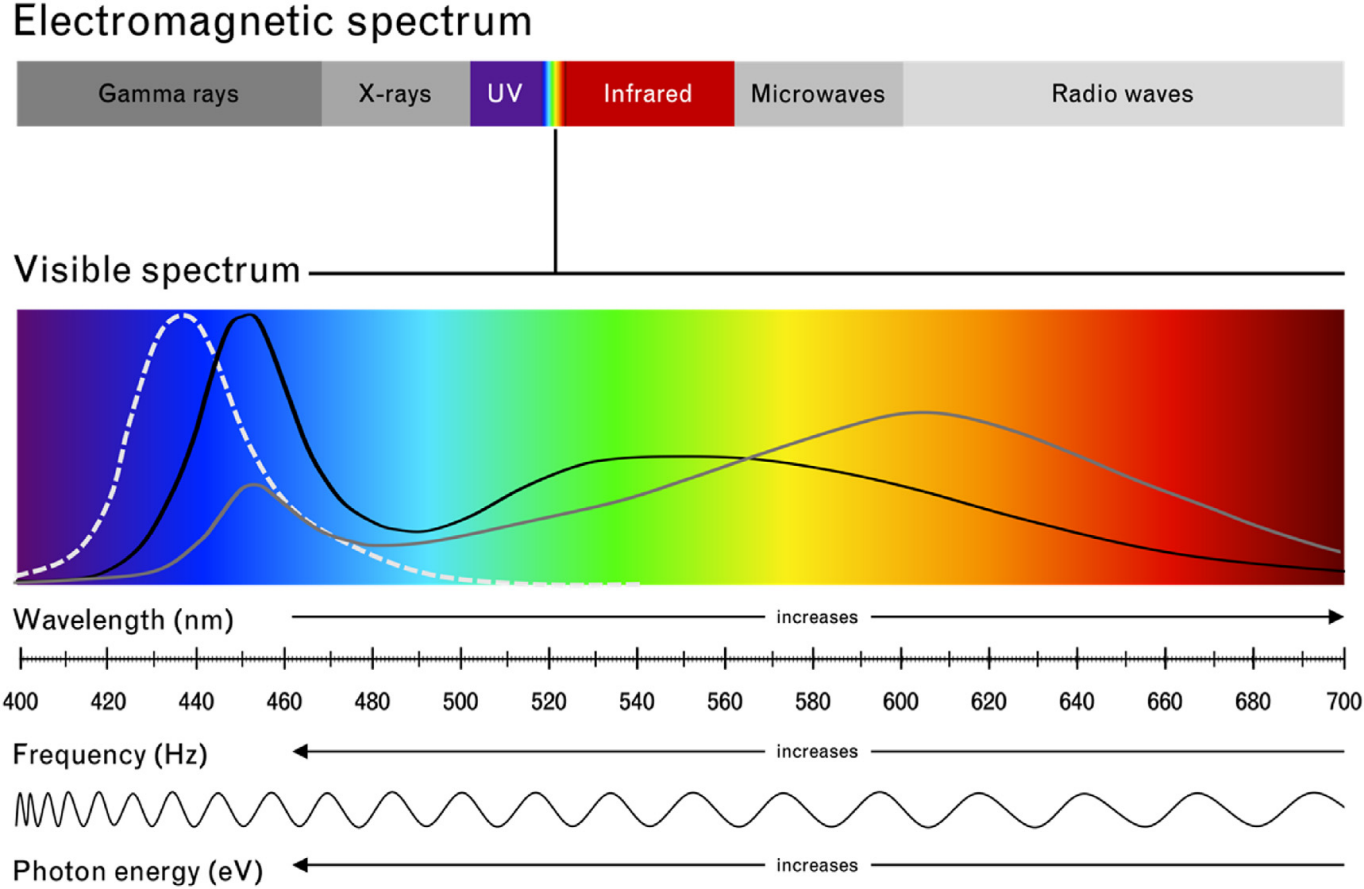
The electromagnetic spectrum. The visible spectrum ranges in wavelength from 400 - 700 nm (nm). As the wavelength increases, the frequency (Hz; Hertz) and photon energy (eV; electronvolt) of light decrease (adapted from [14]). The functions shown on the visible spectrum are approximate visual representations of the blue light hazard function (peak wavelength = 440 nm; dashed line), an emission spectrum of a cold light emitting diode (LED; black line; e.g., peak wavelength for a 6000 Kelvin [K] LED = 455 nm [10]), and an emission spectrum of a warm LED (grey line; e.g., peak wavelength for a 3000 K LED = 600 nm [10]) (adapted from [15]). UV = ultraviolet.

The rapid uptake of personal digital devices over the past decade has exposed us to a new source of artificial light: the illuminated screens of our smartphones, tablets, and similar devices. Today we spend a significant portion of our waking hours in front of digital screens that are illuminated using different types of light-emitting diodes (LEDs). For a digital display to produce a visible image it must either have a backlight or be self-luminous (such as organic LEDs [9]). As a result, the displays of digital devices are necessarily light emitting. LEDs are often used to backlight these displays due to their energy efficiency, durability, and small size [10]. The specific light source used to backlight a display will influence the characteristics of the light emitted from a given digital device [11]. It is common to create polychromatic “white” LEDs by combining a blue LED with a yellow phosphor. This has the effect of producing a light that appears white, but which has a spectral distribution that peaks in the blue portion of the electromagnetic spectrum [12] (Figure 1). The characteristics of the light emitted from digital devices can vary considerably. For a comparison of the spectral peaks and maximum luminance of a range of commercially available electronic displays, please refer to Table 1 in Clark et al. [11]. Given the prolonged periods of time we spend viewing digital devices, often at close range, questions have been raised about the effects the artificial blue light emitted from these devices might have on our health and, in particular, our eyes [13].

There is no doubt that digital devices have become a fundamental part of our day-to-day lives, and technological advancements are unlikely to see them become less prevalent. Yet, there is no consensus in the scientific community on the impact of artificial blue light emitted from digital devices on human health and physiology. As we continue to develop new technologies and light sources that increase our exposure to artificial light, it is relevant to investigate the safety of low-illuminance artificial blue light. This review presents an overview of the current state of research on the blue light safety of digital devices. We begin by describing the properties of blue light and how it is detected by the eye before examining research on the influence of blue light on the eye, visual performance, circadian rhythm, and related processes.

## 2 Blue light and its detection

The eye responds to incoming blue light through its rods, short-wavelength cones, and intrinsically photosensitive retinal ganglion cells (ipRGCs). At the high photon energy end of the visible spectrum, blue light presents a greater risk for harm than other wavelengths of visible light [16]. This risk is what is commonly referred to as the blue light hazard [17]. It is well-established that exposure to visible blue light can cause photochemical damage to the retina and retinal pigment epithelium under certain conditions (e.g., with prolonged exposure or at high intensities) [18]. Photochemical damage is hypothesised to occur when excess absorption of light energy by chromophores in the retina and retinal pigment epithelium causes an overproduction of reactive oxygen species [18, 19, 20]. The exact mechanism behind photochemical damage remains an ongoing area of research [21] and a detailed review of ongoing research in this field is beyond the scope of this review (see Ouyang et al. [21]).

Guidelines for measuring the blue light hazard of artificial light sources, as well as safe viewing limits, have been published by international associations, such as the International Commission on Non-Ionizing Radiation Protection (ICNIRP) [22, 23]. Assessments of the blue light emitted from smartphones and tablets have found that the blue light hazard of these, and similar, digital devices is well below safe viewing limits, even with prolonged exposure [24]. While it is generally agreed that blue light from digital devices does not cause acute (i.e., within a defined period of time after a specific, known exposure timepoint) harm to the retina [11, 24, 25], whether chronic exposure over the lifespan may have a cumulative impact (i.e., a long-term degenerative impact) remains an ongoing area of debate.

The risk blue light poses to the eye is among the chief concerns of the increased use of digital devices that emit artificial blue light. However, artificial blue light also has the potential to influence the circadian rhythm and other processes mediated by the melanopsin system [13]. ipRGCs express the photopigment melanopsin, which preferentially absorbs light in the blue range of the visible spectrum [26]. ipRGCs play a critical role in circadian regulation and participate in various non-circadian processes in the eye and brain [27, 28]. Through the activity of ipRGCs, blue light received by the eye can have far-reaching influences.

## 3 Effects of artificial blue light on the eye

The eye mediates many of the physiological influences of light. Directly exposed to electromagnetic radiation, the eye is uniquely positioned to receive and capture incoming light [29]; however, this also puts ocular structures at a greater risk of light-induced damage. As new artificial light sources are developed, it is pertinent to understand how different light characteristics impact the human eye. The blue component of the emission spectra of LEDs has been of particular concern given the high photon energy of blue light and the widespread application of LEDs in digital device displays [13, 30, 31].

### 3.1 Inflammatory or degenerative effects on the eye surface and refractive ocular adnexae

**Figure 2:**
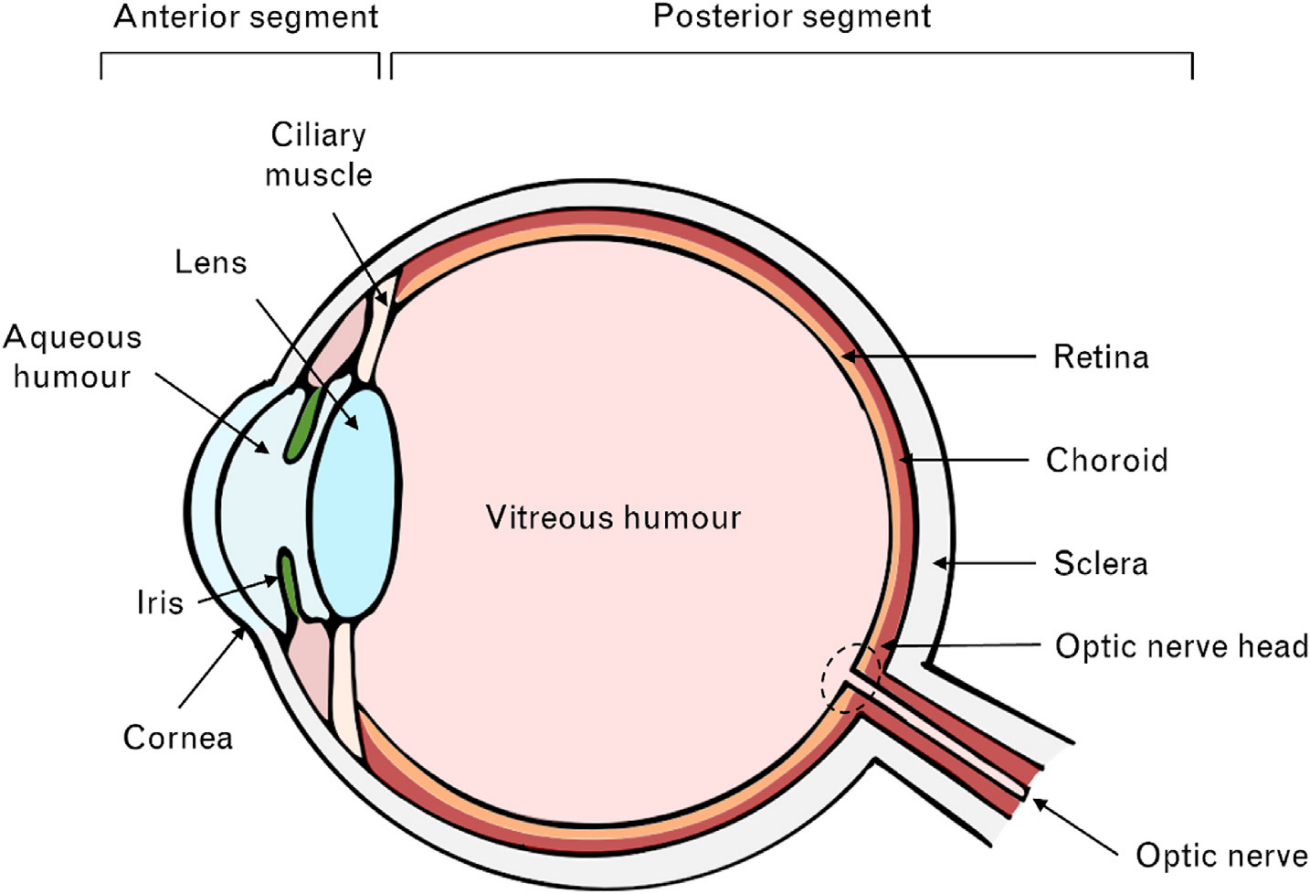
Schematic of the human eye. Key anterior and posterior ocular structures are depicted. Adapted from [32].

To date, much of the concern about the effect of blue light on the eye has focussed on the retina, and scientific understanding of the potential impact of blue light on the ocular surface and other anterior ocular structures remains limited (Figure 2). In mice, overexposure to blue LEDs has been associated with apoptosis and oxidative damage to the cornea [33]. Reduced cell viability and increased production of reactive oxygen species have similarly been observed after exposing human corneal [34] and conjunctival [35] epithelial cells to different wavelengths and intensities of blue light. Such responses can be associated with ocular surface inflammation (see Marek et al. [36] for a discussion of the potential pathways of blue-light-induced ocular surface inflammation), leading some researchers to propose that blue light may provoke or aggravate symptoms of dry eye disease [36]. Yet, the effects of filtering blue light on tear production [37], critical flicker frequency (CFF), and ocular discomfort [38, 39] associated with dry eye disease have been mixed.

The ocular complaints which commonly accompany digital device use have led some to implicate artificial blue light in digital eye strain. However, other factors, such as altered blinking and eye movements, must also be considered when determining the causes of digital eye strain. The evidence for a protective effect of blue-filtering devices against digital eye strain symptoms is also inconsistent [37, 38, 40, 41, 42]. For example, while Cheng et al. [37] found that blue-filtering goggles had no impact on tear production in either control or dry eye participants, filtering blue light did significantly increase perceived ocular comfort among participants with dry eye [37]. In another study, blue light filtering lenses had no significant influence on visual symptoms reported by healthy participants after performing a reading task on a tablet [40]. A blue-blocking screen filter similarly did not alter the perceived visual discomfort of healthy participants performing a digital near work task. The blue light screen filter also did not significantly impact participants’ accommodative responses or pupil dynamics, although it did improve reading speed [41]. Yet, when Lin et al. [38] measured CFF, often used as an objective measure of visual fatigue, they found that participants who wore high blue-filtering spectacles while performing a computer task experienced significantly less eye fatigue (as indicated by a less negative change in CFF) compared to participants in the control and low blue-filtering groups. The high blue-filtering group also reported less ocular discomfort [38]. Still, a further study reported that filtering blue light during a computer task did not significantly alter objective (CFF) or subjective measures of visual discomfort of participants with computer vision syndrome. These results were not influenced by clinician advocacy (positive or negative) prior to the experimental intervention [42]. There is a need for higher-quality research on the potential benefits of filtering blue light for individuals suffering from dry eye disease and digital eye strain, as well as its effect on overall ocular surface health [43].

Over the long term, there is some concern about the potential impact of blue light on the ocular surface of more vulnerable groups, including those suffering from dry eye disease, contact lens wearers, and the elderly [44]. Yet, the experimental setups of existing studies make it difficult to extrapolate the findings to the light conditions experienced when using digital devices. The inconclusive evidence regarding the efficacy of blue-filtering devices on reducing objective and subjective indices of ocular discomfort in both healthy and clinical groups [37, 38, 40] further complicates our understanding of the potential impact, and its quantification, of blue light on the ocular surface.

After passing through the ocular surface, incoming light reaches the aqueous humour and crystalline lens. The crystalline lens absorbs most ultraviolet (UV)-A and UV-B light, as well as some visible blue light [16]. Over the lifespan, as the lens naturally yellows, it absorbs an increasing amount of short-wavelength light [45]. It is hypothesised that low doses of UV light over the lifespan are a risk factor for cataract development. Formation of reactive oxygen species in lens epithelial cells contributes to the development of cataract [46]. It remains to be determined whether visible blue light has a unique and meaningful impact on cataract development beyond the hypothesised role of UV light [30]. Human lens epithelial cells exposed to white LEDs have been found to have reduced cell viability [47] and increased intracellular reactive oxygen species production [48]. Yet, further research is required to understand the significance of these results compared to cumulative exposure of the crystalline lens to natural light over the lifespan, and any additional impact artificial blue light may have on cataract development.

Blue light safety is often discussed in close relation to cataract due to the ongoing debate over blue-filtering intraocular lenses [49, 50]. Traditionally, intraocular lenses inserted during cataract surgery filtered only UV light. It was hypothesised that blue-filtering intraocular lenses would return the eye to a more natural state (as the crystalline lens naturally filters more blue light with age) and offer greater protection against age-related macular degeneration [49]. Yet the benefits of blue-light-filtering intraocular lenses for macular health remain unclear and further research is needed to understand the potential consequences of filtering blue light on other processes (e.g., circadian rhythm) over the long-term [51]. Findings from blue-light-filtering intraocular lens research are referenced at a high level throughout this review. For more in-depth discussions on this topic, please refer to other recent reviews (e.g., Vagge et al. [50]) and meta-analyses (e.g., Downie et al. [52]).

Moreover, melanopsin has also been identified in human crystalline lens epithelial cells and may contribute to the regulation of melatonin synthesis in the lens. Alkozi et al. [53] found that stimulating human crystalline lens epithelial cells with blue light had a selective suppressive effect on melatonin synthesis that was not observed after stimulation with red or green light. This effect was eliminated following administration of a melanopsin antagonist suggesting that melanopsin in the lens is involved in local melatonin synthesis. It is hypothesised that, in addition to that synthesised in the ciliary body, melatonin produced in the lens also contributes to melatonin levels in the aqueous humour. This in turn may influence intraocular pressure (IOP), as melatonin has hypotensive properties in the eye [53]. Initial evidence of this has been reported by Lledó et al. [54] who found that rabbits housed in cages with yellow filters blocking blue light in the 465–480 nm range had lower IOP by up to 43.8 ± 7.8% after three weeks compared to control animals. This reduction in IOP was associated with a significant increase in melatonin at three weeks relative to baseline (16.33 ± 4.50 ng/ml vs. 39.04 ± 5.56 ng/ml). Animals housed under white light conditions exhibited a comparable IOP decrease (32.7 ± 15%) when administered a melanopsin antagonist [54]. Thus, conversely, by stimulating melanopsin in the lens, blue light may influence melatonin production and consequently IOP.

### 3.2 Degenerative effects on neurosensory cells

Beyond the crystalline lens, blue light travels through the vitreous humour to the retina (Figure 2). The ability of visible light to cause photochemical damage to the retina has been known since the 1960s [20] and 1970s [18]. This potential for visible blue light exposure to cause photochemical damage to the retina and retinal pigment epithelium is a concern of the blue-emitting nature of some artificial light sources, such as LEDs [55]. Measurements of the blue light emitted from smartphones, tablets, and computers have consistently been found to be far below the published blue light exposure limits set by ICNIRP. One study found that the blue light spectral irradiance from tablets and smartphones set at maximum brightness ranges from 0.08 to 0.38% of the ICNIRP blue light exposure limit. In comparison, on a sunny day in June, the blue light exposure from viewing the sky in the United Kingdom is approximately 10.4% of ICNIRP's blue light exposure limit [24, 56]. Another study reported that the proportion of blue light emitted from LEDs is not different from that of fluorescent lamps after controlling for the colour correlated temperature, and concluded that LEDs present no special blue light hazard relative to other types of lamps [15]. Clearly, the blue light emitted from digital devices such as smartphones and tablets poses no acute risk to the retina. Whether there is a long-term impact of repeated exposure to low-illuminance artificial blue light remains controversial.

While there are researchers and associations [12, 22, 24, 25, 31, 57] that are of the opinion that chronic exposure to blue light emitted from digital displays is unlikely to have any long-term impact on the retina, other research groups remain concerned. Blue LEDs have been found to reduce cell viability and increase reactive oxygen species production in photoreceptor and retinal pigment epithelium cells [58, 59, 60, 61, 62]. Most research implicating blue light in ocular photodamage has been conducted *in vitro* and in animal models, making it difficult to extrapolate these findings to the potential impact of blue light emitted from digital devices on the human retina and retinal pigment epithelium. These studies urge a more cautious approach to the implementation of LEDs to illuminate our homes and digital devices given that existing standards for safe viewing limits do not consider the cumulative impact of blue light exposure [63].

These studies warn that repeated low-illuminance blue light exposure, such as that from digital displays, may contribute to age-related macular degeneration, which has been attributed to cumulative sun exposure [64]. Yet, it remains unclear to what extent visible blue light contributes to age-related macular degeneration [29, 64, 65]. A meta-analysis found no conclusive evidence that blue-blocking intraocular lenses prevent the development or progression of age-related macular degeneration [52]. These findings suggest that filtering blue light over the long term does not convey a significant retinal health benefit and may be interpreted as blue light lacking a meaningful impact on retinal viability. Overall, the blue light emitted from digital devices does not appear to present a hazard to the retina with chronic exposure; however, there is insufficient evidence to exclude any impact of artificial blue light on the retina over the lifespan.

Finally, incoming light also illuminates the optic nerve head located at the back of the eye (Figure 2). In the absence of light-absorbing chromophores (except the melanopsin-expressing axons of ipRGCs [66]) and cell bodies [67], the optic nerve head may be less susceptible to light-induced damage than the retina. Few studies have investigated the impact of illuminating the optic nerve head with blue light, and while not safety assessments, the paradigms of these experiments suggest that short-term illumination of the optic nerve head with blue light does not pose an immediate (within the duration of the experimental visit) risk of damage [68, 69, 70, 71, 72].

Yet, the inverted design of the human retina positions the axons of retinal ganglion cells in a way that may influence their susceptibility to the damaging effects of light, being unprotected by myelin or macular pigment [73]. The axons of retinal ganglion cells also contain a large number of mitochondria [74], which are vital to cell health and may mediate light-induced damage [75, 76]. To date, studies have found that ipRGCs are less vulnerable to damage and pathologies than other retinal ganglion cell types [77, 78, 79] and maintain their non-image-forming functions after injury [80]. In practice, it is not likely that the interaction of blue light with retinal ganglion cell axons would be damaging to healthy mitochondria [73, 81], but any long-term impact of illuminating the optic nerve head with blue light remains to be determined.

More broadly, blue light also has the potential to influence ocular growth and has been found to inhibit myopia development in some species [82]. In humans, 1 h of blue light exposure reduced the degree of axial elongation associated with hyperopic defocus (defocused eye vs. baseline = -8 μm; non-defocused eye vs. baseline = -6 μm) compared to red and green light [83]. These findings suggest that the protective effects of blue light against myopia may also extend to humans. The mechanism through which blue light influences ocular growth remains unclear. There are some hypotheses that the inhibitory effect of blue light on myopia development may be related to longitudinal chromatic aberration [82], while others have shown an association with dopamine [84] and there is growing evidence that the melanopsin-signalling pathway may also play a role [85]. In children, time outdoors has a protective effect against myopia [86], which is thought to be due to the higher illuminance of outdoor light, but may also be influenced by the spectral composition of sunlight, as it tends to be shifted toward the blue end of the visible spectrum [87]. More research is needed to understand how blue light may influence eye growth and myopia development, as well as the pathways through which it acts.

## 4 The influence of artificial blue light on visual performance

Beyond concerns of light-induced damage to the eye, consideration should also be given to the potential influence of blue light on visual performance. Commonly defined by the speed and accuracy with which visual stimuli can be perceived, key measures of visual performance include visual acuity (i.e., the ability to see small details; sharpness of vision) and contrast sensitivity (i.e., the ability to detect subtle differences in light and dark), among others [88]. A part of the visible spectrum, blue light contributes to image-forming processes. By altering the light environment, artificial light has the potential to impact visual performance. Several older studies using incandescent lamps have measured elevated contrast sensitivity [89] and vernier acuity [90, 91, 92] thresholds under blue illumination versus green, red, yellow, and white light. In addition, there are reports from the late 1980s and 1990s that blue-green flashbacks from argon lasers increase the blue-yellow colour contrast threshold of individuals in some occupations (e.g., ophthalmologists) [93, 94, 95]. Yet, it is unclear whether blue light emitted from digital devices might influence visual performance.

Evidence from blue-light-filtering research suggests that blue light does not contribute significantly to visual acuity or contrast sensitivity [43, 52]. A meta-analysis comparing blue-blocking intraocular lenses to non-blue-blocking intraocular lenses found no clinically meaningful impact of filtering blue light on contrast sensitivity or best-corrected visual acuity [52, 96]. Spectacles that filter blue light have similarly been found not to affect contrast sensitivity [43]; however, there is some evidence that the influence of filtering blue light on contrast sensitivity might depend on an individual's initial contrast sensitivity [97]. Blue-blocking devices also do not appear to significantly affect overall colour vision, although impairments have been reported under mesoscopic conditions in the blue range of the visible spectrum [43, 52, 98, 99, 100]. While further research is required to establish the effect of filtering blue light on visual performance over the long term [43, 49, 52], the existing evidence suggests that blue light alone does not play a central role in these visual processes.

Finally, the blue light emitted from digital devices could influence visual performance by stimulating melanopsin. Melanopsin contributes to spatial pattern representation [101] and contrast sensitivity [102] over a wide range of light intensities [103]. By activating ipRGCs, blue light could also alter retinal dopamine [104], which participates in various retinal processes [105], in particular, retinal light adaptation. Dopamine acts on all major cell types in the retina to adapt retinal signalling over a wide range of light levels [106] with potential functional implications for contrast sensitivity and visual acuity [107]. In the retina, dopamine is also important for circadian entrainment, retinal development, and eye growth [105]. There is a growing body of research demonstrating the involvement of retinal dopamine processes in myopia development [108, 109]. Thus, it remains to be seen how artificial blue light from digital devices might impact visual performance over the long term.

## 5 Artificial blue light, circadian rhythms, and related processes

**Figure 3:**
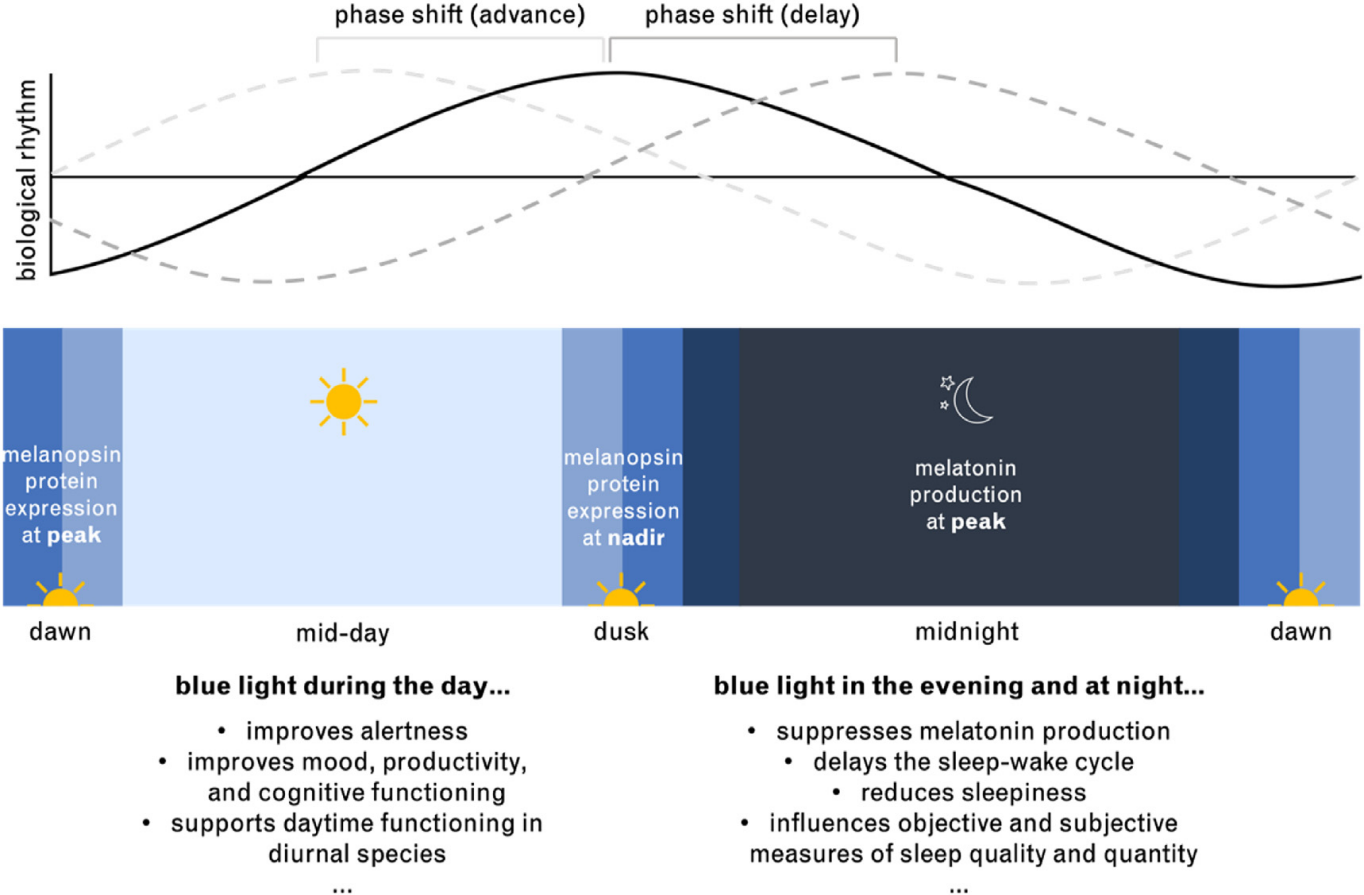
Diagram of the circadian day. Above: A representation of how biological rhythms, which fluctuate throughout the day, can be shifted earlier (phase advanced) or later (phase delayed) by exposure to light [117]. Below: The circadian fluctuations in melanopsin protein expression [118] and melatonin production are depicted. Examples of the influences of blue light exposure during the day, and evening and night are provided [119]. Adapted from [120].

The non-visual influences of blue light on the circadian rhythm (Figure 3) are another concern related to the growing use of digital devices. A circadian rhythm is a biological cycle with a period that is approximately 24 h long. Environmental cues entrain the circadian rhythm to our light-dark cycle. Light is the most important environmental cue, or *zeitgeber*, for circadian regulation, and short-wavelength visible light has the strongest influence on circadian rhythms through ipRGCs [6]. Indeed, melanopsin has been identified as the primary mediator of the non-visual effects of light [110].

Among the non-visual influences of light is the regulation of melatonin, a key circadian neurohormone. Often referred to as the darkness hormone, melatonin is produced at night and suppressed in response to light, and is closely involved in the sleep-wake cycle [6]. Compared to polychromatic and other monochromatic light, blue light has a significantly greater effect on melatonin suppression [111], subjective levels of sleepiness [112], sleep onset [113] and quality [114], and ratio of deep sleep [115]. Even when the photon density of light sources is equal, blue light continues to evoke larger changes in melatonin suppression and alertness [111, 116].

### 5.1 Circadian rhythm

Exposure to blue light during the day is necessary for circadian entrainment and overall well-being [121]. Blue light during the day has beneficial effects on alertness, mood, and productivity [122, 123]. As a result, daytime exposure to blue light from digital devices is unlikely to negatively impact the circadian rhythm. Yet, the effect of blue light on the sleep-wake cycle and circadian entrainment depends on when exposure occurs, according to the phase response curve. In general, early morning exposure to light shifts the circadian rhythm earlier (phase advances), whereas exposure to light in the evening and night shifts the circadian rhythm later (phase delays) and midday exposure has a relatively smaller impact on circadian timing [117]. Blue light is very effective at phase-shifting the circadian rhythm [121, 124] and studies agree that evening exposure to blue light can significantly affect individuals’ sleep-wake cycle [116] and sleep quality [113], especially if the light exposure is chronic [119, 125]. In contrast to the debate surrounding the impact of blue light on the eye, researchers generally agree that repeated exposure to blue light at night has negative consequences on circadian health [121].

There is growing evidence that the blue light emitted from digital devices is sufficient to interfere with sleep when used at night [126]. Delayed sleep onset can occur after evening use of digital devices with screen illuminances as low as 30 lux [113]. Digital device use before bed can also change perceived sleep quality, perceived sleep quantity, and measured sleep efficiency [127]. Other influences [128] may also contribute to the effect of digital device use at night on sleep. It remains to be determined to what extent blue light from digital device use at night affects the sleep-wake cycle and other circadian processes [129], and the best habits to reduce the negative impact of artificial blue light at night. As new LEDs are developed and used to illuminate digital device screens, further research will be needed to investigate their influence on sleep [130].

The negative impact of evening blue light on sleep has led to the development of various blue-filtering devices. Although healthy adults generally do not appear to benefit from wearing blue-blocking spectacles at night [131], there is good evidence that evening blue-blocking spectacle wear reduces sleep onset latency in adults with sleep disorders, jet lag, or who perform shift work [132]. Using blue-blocking devices at night also appears to help manage sleep and circadian rhythm disturbances that accompany certain mood disorders [133]. In contrast to research attributing the poor sleep experienced in older adulthood to the natural filtering of blue light due to the yellowing of the crystalline lens [134], prolonged blue light depletion from blue-blocking intraocular lenses does not appear to impact the sleep-wake cycle [52, 135]; however, more research is necessary [50, 136, 137].

### 5.2 Other physiological and cognitive processes

Blue light has the potential to impact other circadian rhythms beyond the sleep-wake cycle. The eye undergoes rhythmic changes to optimise functioning in response to daily variations in ambient illumination [138, 139, 140]. Altered light rearing environments, including irregular timing of light exposure and chromatic characteristics of light, can change the ocular rhythms in animal models [141, 142] and have been implicated in abnormal eye growth regulation [138, 143]. Blue light has been found to inhibit myopia development and slow ocular growth in some animal models [144] and may explain the protective effect of time outdoors against myopia in children [145].

Exposure to blue light in the evening can also significantly elevate heart rate compared to darkness [146] and equivalent green light exposure [111]. This effect of blue light on heart rate is also observed during the day but does not outlast the stimulus [147]. At night, blue and green, but not red, light suppresses the drop in core body temperature and delays the onset of the temperature decrease after removing the light stimulus [148, 149]. Evening blue light also significantly elevates core body temperature compared to green light [111]. Exposure to LEDs in the evening negatively impacts metabolism by increasing the respiratory quotient and decreasing fat oxidation. A different pattern of results observed after organic LED exposure emphasises the importance of investigating the impact of different light sources and various light characteristics beyond spectral composition [150]. Through the non-visual functions of ipRGCs and the circadian rhythm, blue light likely influences all manner of physiological processes, and when delivered at the appropriate time, is essential for physical and mental health.

Blue light influences mood and cognitive functioning directly, as well as through the circadian rhythm [151]. By synchronising the circadian rhythm to the solar day, blue light indirectly regulates the cognitive states that support daytime functioning in diurnal species. Blue light is also effective at driving transient changes in brain activity. ipRGCs are now known to project to various circadian and non-circadian brain regions [27] and exposure to blue light increases activity in subcortical and cortical structures throughout the brain [152]. Compared to violet light, 50 s of blue light stimulation while performing an n-back task significantly increased the functional magnetic resonance imaging response in the left middle frontal gyrus, left thalamus, and bilateral brainstem of healthy adults [153].

Exposures to blue light, on the scale of several tens of seconds [153] to around 20 min [154] also directly influence cognitive functioning. Blue-enriched light improves performance on tasks requiring sustained attention (e.g., go/no go task) [112] and executive function (e.g., Eriksen Flanker test) [155]. Such improvements may be due in part to the fact that monochromatic blue light significantly increases alertness (reduces sleepiness) compared to equivalent monochromatic green light exposure [111]. In fact, both caffeine and blue light improved accuracy on a visual go/no go task and had an additive effect on the maximum observed decrease in reaction time for participants who received both caffeine and blue light. Notably, when the Eriksen Flanker test was administered, participants exposed to only blue light had significantly better reaction times than those who also received caffeine suggesting that blue light and caffeine have distinct effects on psychomotor responses [155]. Yet, not all studies have measured an improvement in alertness following exposure to blue-shifted light [156].

Light-mediated improvement on certain tasks may also be facilitated by increased concentration, improved ability to think clearly, and reduced mental effort. Office workers exposed to blue-enriched white light had significantly lower mental effort scores (M = 23.67; on a scale from 0-150, 150 representing extreme effort) than their colleagues who worked under white light (M = 26.20) [122]. Students were similarly found to benefit from a greater improvement in concentration when taught and tested in classrooms with blue-enriched white light (pre-test: 181.9 vs. post-test: 216.3; measured using the d2 test) relative to those students who remained in standard lighting conditions (pre-test: 190.5 vs. post-test: 207.8) [157].

Using a blue-light-emitting LED screen has been found to significantly improve declarative memory compared to the use of a non-LED screen with a lower blue emittance [158]. Additionally, daytime exposure to monochromatic blue light has been found to engage brain regions involved in working memory processes, including frontal and parietal regions, to a greater extent than equivalent monochromatic green light [154, 159]. Blue-enriched light has also been found to improve information processing speed. In a classroom setting, children who were taught and tested under blue-enriched light experienced a significant improvement in their cognitive processing speed (pre-test: 2.69 vs. post-test: 3.03), as measured using the German Zahlen-Verbindungs-Test (arbitrary units), compared to their peers who were only tested under blue-enriched light (pre-test: 3.04 vs. post-test: 3.14) or who remained under standard lighting conditions (pre-test: 2.88 vs. post-test: 3.04) [157].

These and other influences of blue light ultimately have the effect of enhancing performance in classrooms. Students taught and tested under blue-enriched light conditions made fewer errors (pre-test: 24.93 vs. post-test: 14.9) than their peers who remained in classrooms with standard lighting (pre-test: 24.1 vs. post-test: 18.30). These benefits extend to workplaces [122, 160] in which blue-enriched lighting conditions lead to more correct responses from night-shift workers on a 1-back task and reduce the number of omission errors on a continuous performance task [160]. In an office setting, blue-enriched light also improves self-reported performance, and increases positive mood compared to standard white light conditions [122]. Appropriately timed, blue-enriched light can also be used therapeutically to improve cognition in some clinical populations, such as Alzheimer's disease. One hour of morning blue light therapy significantly improved participants' Mini-Mental State Examination score after four weeks [161].

On the other hand, prolonged blue light filtering using amber contact lenses resulted in a significant worsening of sustained attention and performance on visuospatial memory tasks in healthy adults [135]. A study in older adults who had undergone cataract lens replacement surgery found that those with UV-filtering intraocular lenses benefited from greater improvements on a procedural learning task after blue-enriched light exposure than age-matched controls and adults with blue-filtering intraocular lenses [162]. Yet, a meta-analysis was unable to conclude with any certainty if blue-blocking intraocular lenses impact daytime alertness or reaction time [52]. Further investigation is required to determine whether there is a significant impact of filtering blue light over the long term on cognitive performance.

## 6 Future directions

As technological advancements continue to proceed at a rapid pace, it is critical to understand how to safely interact with our increasingly digitised world. While the blue light emitted from digital devices is but one consideration when it comes to understanding the safety of new technologies, it remains a contentious, unresolved topic in the scientific community. This review aimed to synthesise and contextualise the available artificial blue light safety research to provide a place from which to begin unravelling the existing inconsistencies in the literature.

There are several challenges to conducting research in the field of artificial blue light safety, not least of which is the inconsistent and varied use of different light measures and terms. In the blue light literature, illuminance (e.g., lux), luminance (e.g., cd/m^2^), irradiance (e.g., W/m^2^), radiance (e.g., W/m^2^-sr), and other units are all used to characterise light sources. This makes it difficult to compare experimental conditions across studies, particularly when other details of the light source are not published and the units cannot be converted. Ultimately, this complicates the synthesis of and expansion on existing research related to the safety of blue light, which may encompass investigations of monochromatic blue, blue-enriched, or cold polychromatic (high colour correlated temperature) light. Moreover, it is also necessary to take into consideration variations between species and ocular structures when extrapolating research findings to the low illuminance artificial blue light exposure humans normally receive. To date, modelling studies have provided insights into the potential long-term effects of repeated artificial blue light exposure and further experimental research will be important to inform future work in this area.

Going forward, it is our opinion that it would be valuable for researchers in this area to establish a more standardised approach to measuring and reporting the characteristics of light sources. To better understand the potential long-term effects of artificial blue light exposure, it may be valuable to conduct longitudinal observational studies that quantify light exposure and measure key indices of ocular health, circadian function, and other metrics of interest. It may be most practical to do so within the scope of an interventional design, for example a study investigating light exposure and myopia inhibition in children. In this way, we would advocate for a multidisciplinary approach to advancing research into the safety of artificial blue light and digital devices given the far-reaching implications of blue light and the rapid pace of technological developments.

A better understanding of how prolonged, repeated exposure to low-illuminance artificial blue light may impact various physiological processes has numerous implications beyond digital device safety. First, it would facilitate the development of new artificial light sources optimally suited to human health and inform how to modify existing light sources, if necessary. It would also support recommendations for good light hygiene practices and healthy device use. Research advancements into the impacts of blue light would contribute to the ongoing debate over the use of blue-blocking intraocular lenses [49, 50] and the contribution of visible light to the pathogenesis of age-related macular degeneration [29, 163].

Further blue light research may help us understand how to leverage artificial light to benefit our health. This may require differentiating between very short visible blue light and “circadian” or “melanopic” blue light. Light therapy is already being used to effectively treat a variety of conditions, in particular, seasonal affective disorder (SAD) [164, 165]. The benefits of light therapy for treating symptoms of SAD are well-established and known to be mediated by the eye [166]. Light therapy for SAD is usually administered for longer periods of time at lower illuminances (e.g., 1–2 h at 1,500–2000 lux) or shorter periods of time at higher illuminances (e.g., 30 min at 10,000 lux) [164]. As the contribution of wavelength to the non-visual influences of light becomes clearer, it is also important to understand how altering the spectral composition of light may impact its therapeutic effects. One study found that narrowband blue light therapy and broadband white light therapy had SAD recovery rates of 77.8% and 85.7%, respectively, over three weeks. This difference was not statistically significant. Importantly, the blue light was administered at 98 lux (λ_peak_ = 464 nm; photon density_424–532 nm_ = 3.35 × 10^14^ photons/cm^2^/s; photon density_380–780 nm_ = 3.38 × 10^14^ photons/cm^2^/s) whereas the white light was delivered at 711 lux (photon density_424–532 nm_ = 3.46 × 10^14^ photons/cm^2^/s; photon density_380–780 nm_ = 7.00 × 10^14^ photons/cm^2^/s), yet both light sources had approximately equal photon densities in the blue range [164]. This finding demonstrates not only that blue light has therapeutic effects (as has been shown previously [165]) but also that these effects are comparable to those associated with white light even at considerably lower illuminances [164]. Blue light therapy is also being used to manage circadian disturbances associated with other conditions, such as Parkinson's disease. One study found that at-home use of blue LED goggles was associated with sleep improvements in 70% of Parkinson's disease participants who reported an effect of the blue light therapy [167]. New insights into artificial blue light could support more effective light therapy practices, including the potential to deliver light therapy using light-emitting digital devices. In the effort to understand the safety of artificial blue light, its potential benefits should not be overlooked.

## 7 Conclusions

In our artificially lit modern lives, our light-emitting smartphones and tablets are among the only light sources we look at directly. As we spend increasing amounts of time engaged with these digital devices, it is relevant to understand how the blue light they emit may impact our health and well-being. While it is generally accepted that the low-illuminance artificial blue light from digital devices has no acute impact on the eye, there remains insufficient high-quality research employing relevant light parameters and exposure conditions to truly understand how the blue light from these devices might impact our eyes over the long term. Findings from blue-light-filtering research suggest that blue light does not significantly impact ocular health, in so far as filtering blue light does not protect against digital eye strain or age-related macular degeneration, however, further research is required [43, 52]. A better understanding of the influences of artificial blue light on human health is crucial to inform how to safely integrate new technological developments into our daily lives. As research continues to investigate the influences of artificial blue light, it will be important to consider not only the potential consequences of ever-evolving technological developments on human health, but also how technology may be used to our benefit.

## Declarations

### Author contribution statement

All authors listed have significantly contributed to the development and the writing of this article.

### Funding statement

This work was supported by the Federal Ministry of Education and Research, Industrie-in-Klinik-Plattform Program BMBF, Germany (13GW0256).

### Data availability statement

No data was used for the research described in the article.

### Declaration of interest's statement

The authors declare the following conflict of interests: Nikita A Wong is supported by Dopavision GmbH. Hamed Bahmani is supported by and has financial interest in Dopavision GmbH.

### Additional information

No additional information is available for this paper.
